# Carbon Allocation in *Rhodococcus jostii* RHA1 in Response to Disruption and Overexpression of *nlpR* Regulatory Gene, Based on ^13^C-labeling Analysis

**DOI:** 10.3389/fmicb.2017.01992

**Published:** 2017-10-11

**Authors:** Martín A. Hernández, Gerd Gleixner, Dirk Sachse, Héctor M. Alvarez

**Affiliations:** ^1^Instituto de Biociencias de la Patagonia, Consejo Nacional de Investigaciones Científicas y Técnicas, Facultad de Ciencias Naturales, Universidad Nacional de la Patagonia San Juan Bosco, Comodoro Rivadavia, Argentina; ^2^Department of Biogeochemical Processes, Max Planck Institute for Biogeochemistry, Jena, Germany; ^3^Section 5.1: Geomorphology, GFZ German Research Centre for Geosciences, Potsdam, Germany; ^4^Institute of Earth and Environmental Science, University of Potsdam, Potsdam, Germany

**Keywords:** *Rhodococcus*, RHA1, NlpR, regulation, ^13^C-glucose, lipid metabolism

## Abstract

Nitrogen lipid regulator (NlpR) is a pleiotropic regulator that positively controls genes associated with both nitrogen and lipid metabolism in the oleaginous bacterium *Rhodococcus jostii* RHA1. In this study, we investigated the effect of *nlpR* disruption and overexpression on the assimilation of ^13^C-labeled glucose as carbon source, during cultivation of cells under nitrogen-limiting and nitrogen-rich conditions, respectively. Label incorporation into the total lipid extract (TLE) fraction was about 30% lower in the mutant strain in comparison with the wild type strain under low-nitrogen conditions. Moreover, a higher ^13^C abundance (∼60%) into the extracellular polymeric substance fraction was observed in the mutant strain. *nlpR* disruption also promoted a decrease in the label incorporation into several TLE-derivative fractions including neutral lipids (NL), glycolipids (GL), phospholipids (PL), triacylglycerols (TAG), diacylglycerols (DAG), and free fatty acids (FFA), with the DAG being the most affected. In contrast, the *nlpR* overexpression in RHA1 cells under nitrogen-rich conditions produced an increase of the label incorporation into the TLE and its derivative NL and PL fractions, the last one being the highest ^13^C enriched. In addition, a higher ^13^C enrichment occurred in the TAG, DAG, and FFA fractions after *nlpR* induction, with the FFA fraction being the most affected within the TLE. Isotopic-labeling experiments demonstrated that NlpR regulator is contributing in oleaginous phenotype of *R. jostii* RHA1 to the allocation of carbon into the different lipid fractions in response to nitrogen levels, increasing the rate of carbon flux into lipid metabolism.

## Introduction

*Rhodococcus* *jostii* RHA1 is an oleaginous bacterium able to accumulate triacylglycerols (TAG) at very high levels with gluconate, glucose, and other carbon sources during growth under nitrogen-limiting conditions or in culture media with a high C/N ratio (Hernández et al., 2008). Oleaginous rhodococcal species are an alternative source for oil production, which can be used by the industry to generate feed additives, cosmetics, oleochemicals, biolubricants, biofuels, or other manufactured products (Alvarez, 2010). The better knowledge on the biology of these microorganisms will make possible the optimization of engineered cells as well as the improvement of technological procedures.

Oleagenicity in rhodococci requires a sophisticated regulatory network for a precise control of the cellular metabolism, involving concerted reactions and pathways in response to diverse environmental signals. This network could be composed by diverse regulatory circuits that execute specific expression programs involved in central and lipid metabolisms. According to previous studies, nitrogen limitation (less than 0.5 g L^-1^ of ammonium chloride or absence of a nitrogen source) is the main condition that triggers lipid accumulation by rhodococci (Alvarez et al., 2000; Voss and Steinbüchel, 2001). In this context, the existence of a putative regulatory mechanism linking nitrogen and lipid metabolisms in these microorganisms is expected.

In a previous study, we identified a regulatory protein called Nitrogen lipid Regulator (NlpR), which contributes to the modulation of nitrogen metabolism, lipogenesis, and TAG accumulation in *R. jostii* RHA1 (Hernández et al., 2017). Under nitrogen-limiting conditions, in which TAG accumulation is stimulated, *nlpR* gene is significantly upregulated, whereas during cultivation of cells under nitrogen-rich conditions its expression is barely detected. NlpR is part of the GlnR regulon, a well-known global regulator of nitrogen metabolism in actinobacteria such as *Rhodococcus, Streptomyces coelicolor* (Amin et al., 2012), *Mycobacterium smegmatis* (Jenkins et al., 2013), *M. tuberculosis* (Williams et al., 2015), *Amycolatopsis mediterranei* (Wang et al., 2013), and *Saccharopolyspora erythraea* (Yao et al., 2014). We demonstrated that the NlpR pleiotropic regulator activates large modules of lipid metabolism by controlling the expression of a set of metabolic genes (Hernández et al., 2017). The predicted NlpR regulon in RHA1 includes several genes involved in NO_3_^-^/NO_2_^-^ assimilation, fatty acid synthesis (FASI and FASII), the Kennedy pathway for TAG and phospholipids (PL) biosynthesis, among others. NlpR binds to a non-canonical motif upstream of the respective genes activating the expression of FASI and diverse genes of FASII involved in FAS as well as *plsC, pap*, and *atf’s* involved in the Kennedy pathway. Thus, this transcriptional regulator seems to modulate large modules of lipid metabolism, regulating the flux within the module when carbon is available. NlpR seems to manage the resource allocation in response to the availability of ammonium concentration in the environment. The central goal of this study was to identify some compounds of the lipid metabolism that are under the influence of this transcription factor in the oleaginous RHA1 strain and to assess how culture conditions impact this regulation. In addition, we analyzed the possible impact of NlpR on sugar-derived compounds. For this, we investigated two different genetic-modified strains of RHA1 specifically related to NlpR transcriptional regulator to understand the role of this protein within an integrated metabolic context. The disruption of *nlpR* gene under nitrogen-limiting conditions, in which this regulator is usually induced, could show the impact of this protein on the biosynthesis of the different lipid species as well as other compounds. On the other hand, overexpression of *nlpR* gene under nitrogen-rich conditions, in which this regulator is natively not induced, could artificially force the activation of targeted genes involved in lipogenesis, confirming its impact on lipid metabolism of oleaginous rhodococcal cells. Regulator-induced flux changes in the two engineered strains were further validated by isotopic-labeling experiments to determine ^13^C distribution among final products produced by cells under N-limiting and N-rich conditions, respectively. Beyond discovery of novel regulatory interactions in metabolism, this study provided further insights into transcriptional regulation of metabolism in the oleaginous *R. jostii* RHA1.

## Materials and Methods

### Strains and Culture Conditions

The strains used in this work are listed in **Table 1**. All *Rhodococcus* strains were cultivated aerobically at 30°C in 20 mL of LB media from their respective isolates colonies on LB agar plates. From these cultures cells, a second inoculum was transferred and cultured in a 50 mL of fresh LB medium overnight at 30°C and 200 rpm. Cells were harvested and washed with a sterile NaCl solution (0.85%, w/v). The pellets were resuspended with the same solution and inocula with similar biomass were transferred to 300 mL of minimal salt medium (MSM) (9 g L^-1^ Na_2_HPO_4_.12H_2_O, 1.5 g L^-1^ KH_2_PO_4_, 0.1 g L^-1^ NH_4_Cl, 0.2 g L^-1^ MgSO_4_.7H_2_O, 20 mg L^-1^ CaCl_2_.2H_2_O, 1.2 mg L^-1^ Fe(III)NH_4_-Citrate, and 0.1 ml L^-1^ of trace elements solution SL6 [10 mg L^-1^ ZnSO_4_.7H_2_O, 3 mg L^-1^ MnCl_2_.4H_2_O, 30 mg L^-1^ H_3_BO_3_, 20 mg L^-1^ CoCl_2_.6H_2_O, 1 mg L^-1^ CuCl_2_.2H_2_O, 2 mg L^-1^ NiCl_2_.6H_2_O, 3 mg L^-1^ Na_2_MoO_4_.2H_2_O] in distilled water). The MSM media were supplemented with 0.1 g L^-1^ (in the case of RHA1 WT and RHA1::*nlpR* strains) or 1 g L^-1^ (in the case of RHA1 pTipQC2 and RHA1 pTipQC2/*nlpR* strains) of ammonium chloride as nitrogen source. Glucose was used in these MSM media as sole carbon source at a final concentration of 0.5% (w/v) as a mix of unlabeled glucose (90%) and labeled glucose [10%, (1-^13^C1)-glucose, Sigma-Aldrich]. Cells were harvested at the end of the exponential phase, washed with a sterile NaCl solution (0.85%, w/v), and freeze-dried for chemical analyses. Antibiotics were used at the following final concentrations: 50 μg/mL kanamycin (Km) and 34 μg/mL chloramphenicol (Cm). For overexpression analysis of *nlpR* gene under the thiostrepton promoter (*PtipA*) of pTipQC2, a final concentration of 5 μg/mL of thiostrepton was added to cell cultures at time 0.

**Table 1:** Strains used in this study.

Strain	Description	Culture conditions	Reference
RHA1 WT	**Wild type (WT) strain**	MSM0.1	Seto et al., 1995
RHA1::*nlpR*	**Mutant strain** (RHA1 strain with *nlpR* gene disruption, Km^R^)	MSM0.1	Hernández et al., 2017
RHA1 pTipQC2	**Control strain** (RHA1 strain containing the empty pTipQC2 vector. Cm^R^, Thio^R^)	MSM1	Hernández et al., 2017
RHA1 pTipQC2/*nlpR*	**Overexpressing strain** (RHA1 strain containing the pTipQC2/*nlpR* vector. Cm^R^, Thio^R^)	MSM1	Hernández et al., 2017

### Lipid Extraction and Fractionation Methods

Total lipid extracts (TLEs) were obtained from same dry biomass (100 mg) for each bacterial strain with 50 mL of the solvent mix dichloromethane (DCM) and methanol (9:1 v/v) and ultrasonic bath for 2 h at room temperature. Samples were then filtrated, fully dried, resuspended in a small volume (4 mL of DCM) and stored for further analysis. Fractionation of 25% of each TLE was carried out by solid-phase extraction (SPE) column chromatography containing 2 g of unmodified silica stationary phase (Chromabond^®^ SiOH, Macherey-Nagel) after Kramer and Gleixner (2008). Fractions collected were as follows: NL (1 Volume, chloroform), Glycopids Gly (1 Volume, acetone), and PL (4 Volume, methanol). Each fraction was fully dried, resuspended in 2 mL of chloroform, and analyzed by TLC to identify the corresponding compounds of interest. As expected, positive spots were detected in fractions of glycolipids (GL), PL, and neutral lipids (NL), the last one showing several species such as TAG, DAG, and monoacylglycerols (MAG) (Supplementary Figure S1A). It is important to consider that GL and PL fractions may be likely underestimated and might be a mix of GL and PL compounds, according to Heinzelmann et al. (2014).

Alternatively, fractionation of 50% of each TLE was carried out by SPE column chromatography containing 1.5 g of an aminopropyl stationary phase modified after Sessions et al. (1999) with some modifications. This time, samples were eluted in five fractions: F1 (10 mL hexane, usually used for hydrocarbons and esters extraction), F2 (10 mL hexane/DCM 4:1, esters and ketones), F3 (10 mL DCM/acetone 9:1, alcohols), F4 (10 mL DCM/formic acid 4:1, acids), and F5 (10 mL DCM/methanol 1:1, remaining acids). All fractions were fully dried, resuspended in 3 mL of DCM, and analyzed by TLC to identify compounds of interest. Interestingly, no spots were detected in F1 and F4 by TLC analysis, but were found in F2 (TAG), F3 (TAG+DAG), and F5 (FFA) (Supplementary Figure S1B). In order to analyze the TAG and DAG separately, 1.1 mL F3 was fractioned in two new fractions by elution first with hexadecane/DCM and then with DCM/acetone using an aminopropyl stationary phase–based column yielding in F3a and F3b (Supplementary Figure S1C). Both fractions were dried and resupended in 1.1 mL of chloroform. Fractions GL, PL, NL, F2+F3a, F3b, and F5 were chosen for further analysis and referred as GL, PL, NL, TAG, DAG, and FFA, respectively.

### Lipid Analysis

Identification analyses of TLE-derivative lipid fractions from *Rhodococcus* cells were carried out by thin-layer chromatography (TLC). A total of 14 μL of each fraction were subjected to TLC on silica Gel 60 F254 plates (Merck) using hexane/diethyl ether/acetic acid (80:20:1, v/v/v) as solvent lipids analysis (Wältermann et al., 2000).

Selected TLE-derivative fractions (F2, F3, F3a, F3b, F5, NL, and PL) were hydrolyzed and methylated using KOH/methanol solution resulting in the corresponding fatty acids methyl esters (FAMEs). Resultant FAMEs were purified using a chromatography column containing 0.5 g of aminopropyl-modified silica stationary phase (Chromabond^®^ NH_2_, Macherey-Nagel), dried under a nitrogen stream, and resuspended in a 300 μL of a stock solution of tridecanoic acid methyl ester in isooctane (500 ng/μL for F2, F3, F3a, and NL; 100 ng/μL for F3b, F5, and PL), used as internal standard. The FAME content of all fractions was analyzed on a GC-FID 7890B with a programable temperature vaporization (PTV) injector (Agilent Technologies, Palo Alto, CA, United States) using a DB-1MS UI column (60 m × 0.25 mm internal diameter × 0.25 μm film thickness, Agilent Technologies, Palo Alto, CA, United States) and helium as carrier gas (1.8 ml/min). The temperature program started with 45°C for 1 min, then increased in a first ramp of 60°C/min to 140°C (held for 0.5 min), followed by a second ramp of 2°C/min until 264°C and a third ramp until 320°C (held for 3 min). Directly after injection, the PTV was heated up from 55 to 280°C at a rate of 500°C/min. Compound-specific ^13^C isotope analysis of FAMEs was done by GC-IRMS (GC 7890A with PTV injector, Agilent Technologies, Palo Alto, CA, United States; coupled via a Conflo IV/GC IsoLink to a Delta V Plus IRMS, Thermo Fisher Scientific, Bremen, Germany) using a DB-1MS UI column (60 m × 0.25 mm internal diameter × 0.25 μm film thickness, Agilent Technologies, Palo Alto, CA, United States) and helium as carrier gas (1.8 ml/min). Directly after injection, the PTV was heated up from 55 to 280°C at a rate of 500°C/min. The GC temperature program started with 45°C for 1 min, then increased in a first ramp of 60°C/min to 140°C (held for 0.5 min), followed by a second ramp of 4°C/min until 283°C (held for 4.9 min) and a third ramp until 320°C (held for 3 min). Concentrations and ^13^C isotope content of identified FAMEs were corrected for the methyl group introduced during derivatization.

^13^C analysis of cellular biomass and ^13^C analysis of dried lipid fractions were performed on an elemental analyzer coupled to an isotope ratio mass spectrometer [EA-IRMS model CE1100 coupled on line via a Con FloIII (27) interface with a Delta^+^ isotope ratio mass spectrometer; all supplied by Thermo Fisher Scientific, Germany]. For this, 0.1 mg of solid samples or 40 μL of liquid lipid fractions were placed into tin capsules (liquid samples were fully dried); and combusted in oxygen stream of the elemental analyzer and evolved CO_2_ was transferred to the isotope ratio mass spectrometer to determine ^13^C values.

### Glycogen and Extracellular Polymeric Substance (EPS) Extraction and Analysis

Glycogen was extracted as previously described in Hernández et al. (2008) from dried samples (approximately 50 mg) and processed similarly to starch according to Richter et al. (2009), with some modifications. The obtained pellets after glycogen purification were washed two times with water (HPLC-grade water) and centrifuged at 10,000 g for 5 min. The samples were then dried at 60°C in a drying oven. Samples were resuspended in 500 μL of 50 Mm of sodium acetate buffer (pH 5), then 250 μL of purified α-amylase (3000 U mL^-1^), and 250 μL of amyloglucosidase (2500 U mL^-1^) solution were added to each sample and incubated at 55°C for 2 h. A blank (500 uL buffer and 250 μL amylase and 250 μl of amyloglucosidase) was also prepared and treated as the samples. To separate the enzyme proteins from the glycogen hydrolysates, the blank and samples were ultrafiltrated through pre-cleaned centrifugal filter devices (Microcon YM-10, Regenerated Cellulose, 10,000 Da MWCO, Sartorious, Göttingen, Germany) at 12,000 g for 30 min. The retentate was discarded and 100 μL of the filtrate was pipetted into smooth tin capsules and dried before isotope analysis by EA-IRMS.

Extracellular polymeric substance (EPS) production was monitored by observation of cells by optic microscopy from total cell cultures and total sugar quantification by means of the phenol–H_2_SO_4_ method from supernatant cell cultures. EPS was purified from MSM0.1 culture media containing RHA1 wild type (WT) cells or mutant cells. For this, portions of cell cultures containing a same cellular biomass were vigorously vortexed and then centrifuged at 11,000 rpm for 40 min. Supernatants were filtrated in 0.45 μm filters and then mixed with 3 volumes of cold ethanol. The mixtures were stored overnight at 4°C and after centrifuged at 11,000 rpm for 40 min. The resultant pellets were then resuspended in HPLC-grade water and dialyzed overnight with distilled water with a 10K MWCO membrane. Then, samples were mixed again with 3 volumes of cold ethanol. The mixtures were stored overnight at 4°C and after centrifuged at 11,000 rpm for 40 min. The resultant pellets were then washed twice with 70% cold ethanol and lyophilized. Dried samples were resuspended in water and 10 μL were pipetted into smooth tin capsules and dried before isotope analysis by EA-IRMS.

## Results

### Cultivation of RHA1 Cells under Nitrogen-Low and Nitrogen-Rich Conditions and Isotopic Signature of Cellular Biomass

The ^13^C assimilation into bulk biomass, lipid, and carbohydrate (glycogen and EPS) fractions of RHA1 derivative strains (**Table 1**) was traced by using ^13^C-labeled glucose (in addition to non-labeled glucose) as carbon and energy source. The wild type strain (RHA1 WT) and the *nlpR*-disrupted mutant strain (RHA1::*nlpR*) were cultivated in MSM0.1 medium (nitrogen-limiting conditions) to stimulate lipid accumulation, whereas the control strain (RHA1-pTipQC2) and the *nlpR*-overexpressing strain (RHA1 pTipQC2/*nlpR*) were grown in MSM1 medium (nitrogen-rich conditions), which stimulates growth but not lipid accumulation. Cells were harvested at the end of the exponential growth phase in order to investigate the incorporation of ^13^C into cellular biomass and cellular fractions (Supplementary Figure S2). The resultant biomass yields, biomass^13^C content, and the ^13^C use efficiency for each strain are shown in **Table 2**. Whereas the substrate uptake efficiency between parental and mutant strains grown on glucose was similar, a slight decrease in the uptake efficiency in the overexpressing strain compared to the control was observed, with a concomitant slight decrease in the biomass yield (**Table 2** and Supplementary Figure S2). Since both control and overexpressing strains were cultivated under identical growth conditions, including antibiotics or inducers, this effect could be attributed to the induced *nlpR* expression in cells.

**Table 2:** Biomass, biomass^13^C content, and the ^13^C use efficiency in strains analyzed in this work based on EA-IRMS analysis.

Strain	Biomass (mg)	Biomass ^13^C content (mg)	^13^C use efficiency (%)^∗^
RHA1 WT	358.8 ± 7.3	5.1	30.8
RHA1::*nlpR*	341.3 ± 17.1	4.6	27.7
RHA1 pTipQC2	475.8 ± 41.2	6.7	40.1
RHA1 pTipQC2/*nlpR*	322.8 ± 6.0	5.2	31.1

*^∗^Calculated as (mg ^*13*^C microbial biomass/^*13*^C Sugar added) × 100.*

### Isotopic Signature of TLE, EPS, and Glycogen

**FIGURE 1:**
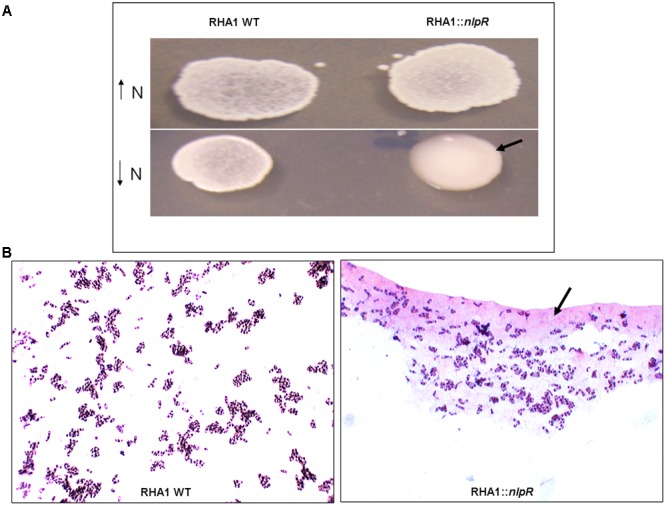
Growth of wild type (RHA1 WT) and mutant (RHA1::*nlpR*) strains on solid media. **(A)** Cells were grown in MSM1 solid medium (high nitrogen content) and MSM0.1 solid medium (low nitrogen content). The mutant strain with mucoid phenotype is highlighted with an arrow. **(B)** Optic microscopy of RHA1 **(Left)** and RHA1::*nlpR* cells **(Right)** from colonies grown under low-nitrogen conditions. The EPS around mutant cells is highlighted with an arrow.

To explore the contribution of NlpR on regulation of lipid metabolism, we analyzed the assimilation of ^13^C-enriched glucose into the TLE and its derivate fractions, based on loss and gain of *nlpR* expression, under nitrogen-limiting and nitrogen-rich conditions, respectively. Since colonies of the mutant strain exhibited a mucoid appearance and an abundant extracellular material occurred around the corresponding cells after cultivation under nitrogen-limiting conditions (**Figures 1A,B**), we also extended the analysis of assimilation of ^13^C-enriched glucose into the sugar-related fractions (glycogen and EPS) in both mutant and WT strains cultured in a nitrogen-poor medium. Unlabeled quantification analyses of total lipids and carbohydrates revealed approximately a 1.7-fold decrease in total fatty acid (FA) content, 3.5-fold increase in glycogen, and 5.7-fold increase in EPS fractions in mutant strain RHA1::*nlpR* compared with the WT strain (**Table 3**). Interestingly, these physiological changes were not observed after cultivation of cells under nitrogen-rich conditions, which indicates that nitrogen deprivation is the trigger for the appearance of *nlpR*-dependent phenotypes as it was suggested in our previous work (Hernández et al., 2017). In addition, no changes in colonies aspect were observed with *nlpR*-overexpressing cells in comparison with control cells during cultivation under N-limiting as well as N-rich conditions (data not shown).

**Table 3:** Quantification of total lipid, glycogen, and EPS in RHA1 WT and RHA1::*nlpR* mutant strain grown under nitrogen-low conditions (MSM0.1).

Strain	Total lipid^a^ (%CDW)	Glycogen^b^ (%CDW)	EPS^c^ (μg/mL)
RHA1 WT	54.3 ± 3.6	0.6	8.65
RHA1::*nlpR*	31.3 ± 2.1	2.1	49.7

*^a^Measure as % of total fatty acids content by cellular dry weight (CDW) by conventional GC analysis. ^b^Measure as free glucose obtained by enzymatic hydrolysis. ^c^Measure as total sugars by phenol–H_*2*_SO_*4*_ method in free cells supernatants from MSM0.1 culture media.*

**FIGURE 2:**
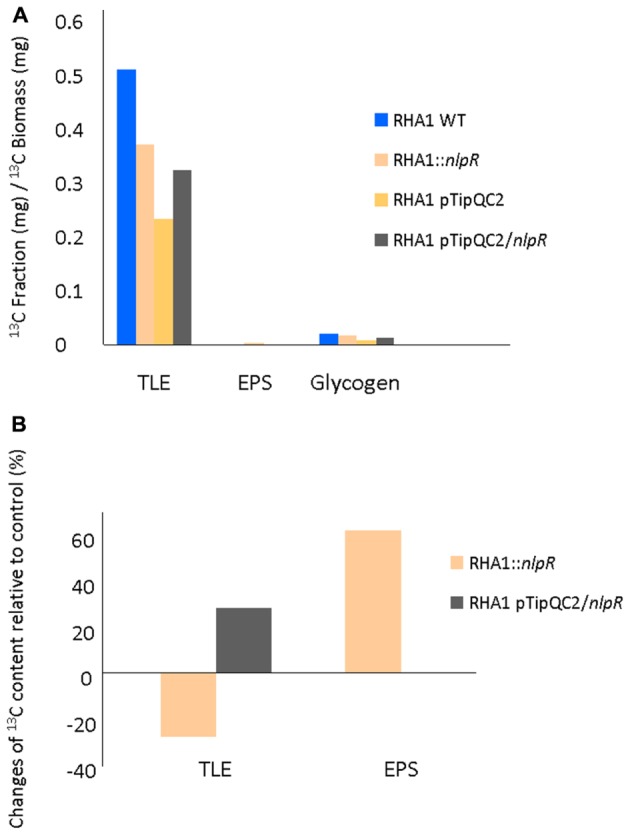
^13^C abundance analysis in RHA1-derivative strains based on EA-IRMS analysis. **(A)** ^13^C content in TLE, EPS and glycogen normalized by ^13^C biomass in both mutant and overexpressing strains compared to the wild type (WT) and control strain, respectively. **(B)** Changes in ^13^C content in TLE and EPS fractions of mutant strain compared to the WT strain and in TLE of overexpressing strain compared to the control strain. WT and mutant cells were grown in nitrogen-low conditions, whereas control and overexpressing cells were grown in nitrogen-rich conditions.

Quantitative inspection of the labeling data by EA-IRMS analysis, revealed that the ^13^C abundance in the TLE normalized by the cellular biomass was lower in the mutant strain compared to the WT strain (**Figure 2A**). On the other hand, when cells were cultivated under nitrogen-rich conditions, in which *nlpR* gene is usually not induced, a higher ^13^C incorporation was observed in the TLE of overexpressing strain in comparison with the control strain (**Figure 2A**). Although absolute values of ^13^C incorporation in the EPS fraction were very low in both WT and mutant strains compared to the TLE, a higher resolution analysis of this fraction revealed a significant increase of ^13^C abundance (∼60%) in the EPS fraction of the mutant strain relative to WT strain (**Figure 2B**), even higher than TLE fraction. Finally, we also analyzed the dynamics of glycogen contents in cells. Despite the fact that the absolute values of quantified glycogen were higher in the mutant strain compared to the WT (**Table 3**), no significant changes in ^13^C incorporation to this compound was observed between both strains (**Figure 2A**).

### ^13^C Analysis of TLE-Derivative Fractions

From the TLE fractions of each investigated strain, several fractionation steps were performed in order to obtain fractions containing different lipid compounds, such as GL, PL, and NL, the last ones composed by TAG, DAG, and MAG (Supplementary Figure S1).

**FIGURE 3:**
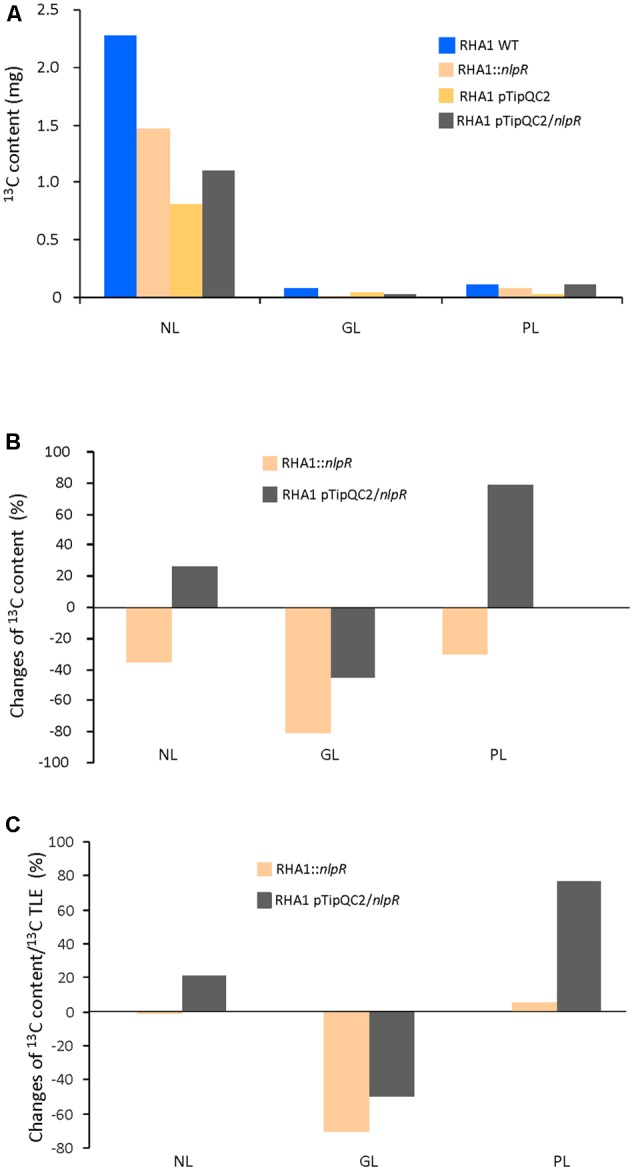
^13^C analysis in NL, PL, and GL fractions in RHA1-derivative strains based on EA-IRMS analysis. **(A)** Absolute ^13^C content in NL, GL, and PL fractions in mutant and overexpressing strains compared to the wild type (WT) and control strain, respectively. **(B)** Changes in ^13^C content in NL, GL, and PL fractions in mutant and overexpressing strains compared to the WT and control strain, respectively. **(C)** Changes of ^13^C content in NL, PL, and GL fractions in mutant and overexpressing strains based on ^13^C content of their TLE fractions and compared to the WT and control strain, respectively. WT and mutant cells were grown in nitrogen-low conditions, whereas control and overexpressing cells were grown in nitrogen-rich conditions.

As expected, ^13^C abundance in NL fraction of all strains was higher than in GL and PL fractions as is shown in **Figure 3A**. EA-IRMS analysis revealed that absolute ^13^C incorporation in NL, GL, and PL fractions was lower in the mutant strain compared to the WT strain (**Figure 3A**). On the other hand, absolute ^13^C abundance in the NL and PL fractions was higher in overexpressing cells compared to the control cells (**Figure 3A**). A similar tendency in ^13^C abundance in the NL or PL between mutant vs WT strain and overexpressing cells vs control cells was observed after GC-IRMS analysis (Supplementary Figures S3A,B). The relative changes of ^13^C contents for each lipid fraction relative to their respective controls in both mutant and overexpressing strains are shown in **Figure 3B**. Interestingly, the ^13^C enrichment of NL relative to TLE was similar between the disrupted mutant and the WT but higher in the NL and PL fractions of the *nlpR*-overexpressing strain in comparison with its control. Finally, a decrease of ^13^C enrichment in GL relative to TLE occurred in both mutant and overexpressing strains cultivated under nitrogen-limiting and nitrogen-rich conditions, respectively (**Figure 3C**).

### ^13^C Analysis of NL-Derivative and FFA Fractions

**FIGURE 4:**
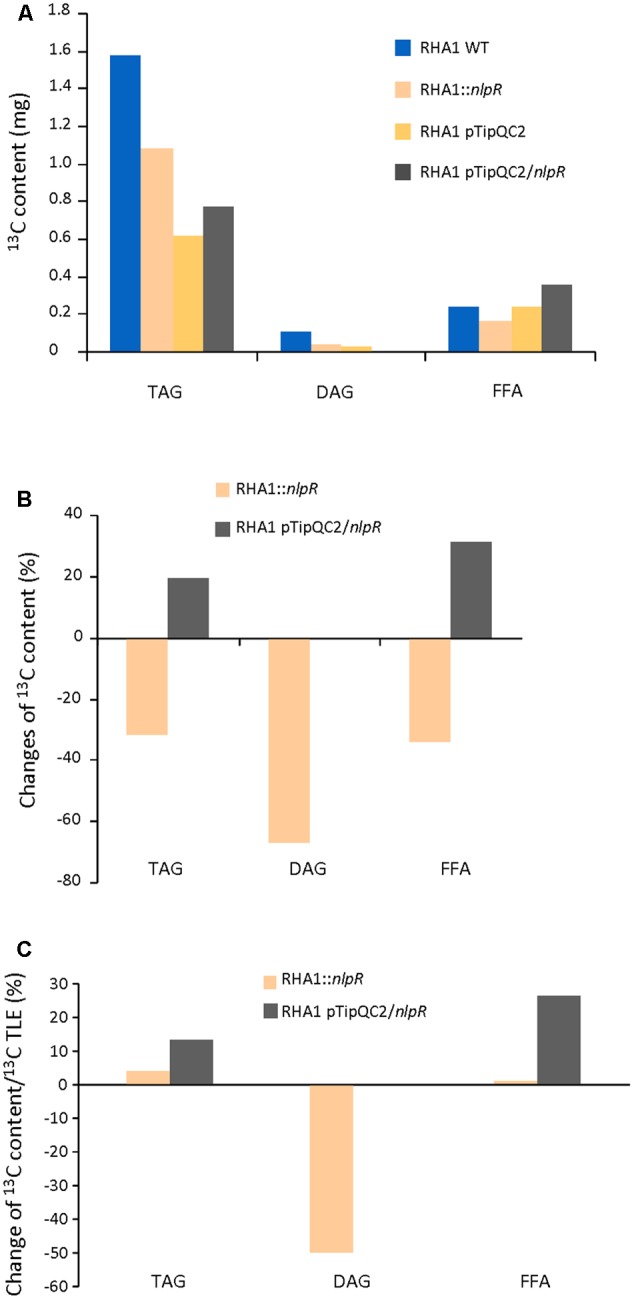
^13^C analysis in TAG, DAG, and FFA fractions in recombinant strains based on EA-IRMS analysis. **(A)** Absolute ^13^C content in TAG, DAG, and FA in mutant and overexpressing strains compared to the wild type (WT) and control strain, respectively. **(B)** Changes in ^13^C content in TAG, DAG, and FFA fractions in mutant and overexpressing strains compared to the WT and control strain, respectively. **(C)** Changes in ^13^C content in TAG, DAG, and FFA fractions in mutant and overexpressing strains based on ^13^C content of their TLE fractions and relative to the WT and control strain, respectively. WT and mutant cells were grown in nitrogen-low conditions, whereas control and overexpressing cells were grown in nitrogen-rich conditions.

We analyzed the ^13^C incorporation in both NL-derivative (TAG and DAG) and FFA fractions by EA-IRMS. The ^13^C abundance in TAG fraction of all investigated strains was significantly higher than in DAG and FFA fractions, as is shown in **Figure 4A**. The absolute values of ^13^C incorporation into TAG, DAG, and FFA were lower in the mutant compared to the WT (**Figures 4A,B**). The *nlpR* disruption specially affected the incorporation of labeled carbon to DAG fraction, as is shown in **Figure 4B**. In addition, the ^13^C enrichment of DAG fraction relative to TLE was largely decreased in the case of the disrupted mutant strain in comparison with the WT strain (**Figure 4C**). Similar results were obtained when ^13^C abundance in TAG, DAG, and FFA sub-fractions of mutant and WT strains was measured by GC-IRMS analysis (Supplementary Figures S3C–E).

On the other hand, EA-IRMS analysis revealed that overexpression of *nlpR* gene promoted an increase of ^13^C abundance in TAG and FFA fractions, during cultivation of cells under nitrogen-rich conditions as is shown in **Figures 4A–C**. In addition, GC-IRMS analysis confirmed also the increase of DAG fraction, in addition to TAG and FFA (Supplementary Figure S3C–E). Thus, an increase of ^13^C abundance occurred in all these lipid fractions in RHA1 pTipQC2/*nlpR* strain compared to control cells.

### Fatty Acid (FA) ^13^C Isotopic Composition for NL, PL, TAG, DAG, and FFA Fractions

**FIGURE 5:**
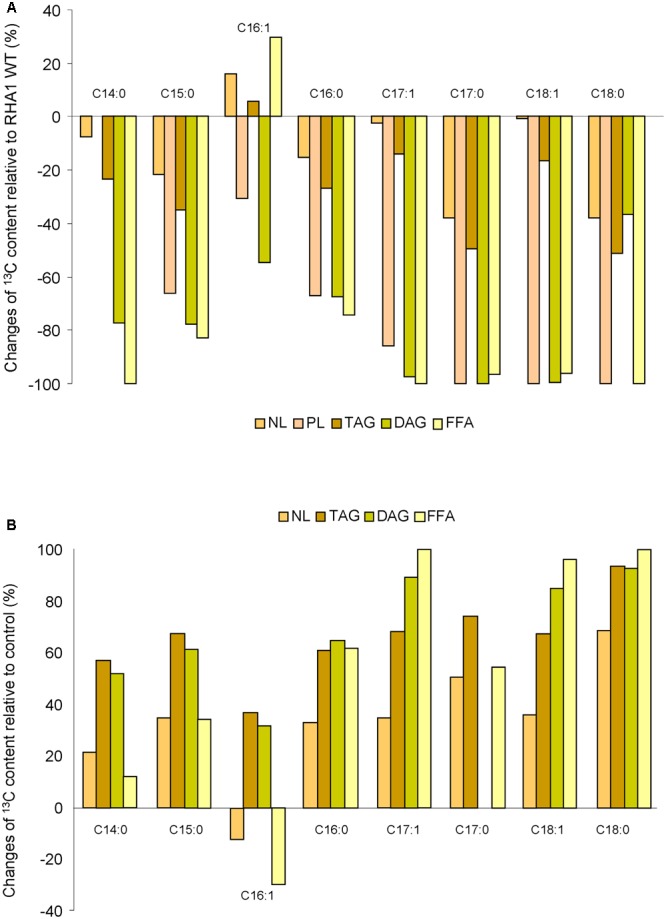
Changes of ^13^C content on individual FA species based on GC-IRMS analysis in several lipid fractions of mutant strain **(A)** and overexpressing strains **(B)** relative to the wild type (WT) and control strain, respectively. WT and mutant cells were grown in nitrogen-low conditions, whereas control and overexpressing cells were grown in nitrogen-rich conditions.

To analyze the FA ^13^C isotopic composition in NL, PL, TAG, DAG, and FFA, all fractions were derivatized and analyzed by GC-IRMS. Whereas no qualitative differences in the FA profiles were observed between the WT and RHA1::*nlpR* strain, the ^13^C absolute content of all FAs were lower for all analyzed fractions (NL, PL, TAG, DAG and FFA) in the mutant strain (Supplementary Figure S4). However, palmitic acid (C_16:0_), heptadecenoic acid (C_17:1_), heptadecanoic acid (C_17:0_), and oleic acid (C_18:1_) in the NL and TAG fractions and palmitic acid (C_16:0_) in the PL, DAG, and FFA fractions were still the main FAs found in the mutant strain (Supplementary Figure S4). **Figure 5A** summarizes the changes of each FA species in the analyzed lipid fractions of mutant strain relative to WT strain. The sum of the individual FA was in accordance with the lower ^13^C enrichment of the corresponding lipid fractions analyzed by EA-IRMS. On the other hand, the ^13^C absolute content of the majority species of FA was higher in the NL, TAG, DAG, and FFA fractions in the overexpressing cells in comparison with control cells, during cultivation under nitrogen-rich conditions (Supplementary Figure S4). The expression of *nlpR* gene under these conditions seemed to produce larger changes on C_17:1_, C_18:0_, and C_18:1_ species in TAG, DAG, and FFA fractions (**Figure 5B**). Interestingly, the ^13^C abundance variation of palmitoleic acid (C_16:1_) in the disrupted mutant and in the overexpressing strains behaved in the opposite way in regard to the rest of the FA species for NL and FFA fractions (**Figures 5A,B**).

## Discussion

### The Role of *nlpR* under Nitrogen Limitation

NlpR seems to be an important regulatory component to control nitrogen-mediated carbon distribution to lipogenesis in *R. jostii* RHA1. In this study, we compared the mutation and overexpression effects of the *nlpR* gene by using ^13^C-labeled glucose to analyze the contribution of the NlpR protein in the carbon metabolism of this oleaginous bacterium. Results of this study revealed that the alteration of *nlpR* expression in cells (by gain or default of gene expression) altered the metabolic isotope allocation into different carbon pools during cultivation under nitrogen-limiting and nitrogen-rich conditions.

Since *nlpR* expression is induced by nitrogen limitation and is thought to be involved in lipid metabolism (Hernández et al., 2017), we analyzed the effect of *nlpR* gene disruption on the distribution of the labeled carbon through the metabolism during cultivation of cells under nitrogen-limiting conditions. The *nlpR* disruption promoted a decrease of carbon fluxes to lipogenesis, and a partial redirection of output fluxes to sugar-derived products such as glycogen and EPS. The increase of glycogen content has been also reported for rhodococci when lipid accumulation was prevented by mutations or the addition of cerulenin (Hernández et al., 2008, Hernández and Alvarez, 2010). Surprisingly, despite a higher amount of glycogen accumulated by the mutant strain, no significant changes in ^13^C abundance occurred in the glycogen fraction of the mutant compared to the WT. This result may suggest that glycogen synthesis in the mutant predominately utilized phosphorylated sugars originated through the gluconeogenic pathways, instead of directly using the labeled ^13^C-glucose utilized as substrate for cell cultivation. Isotope labeling experiments showed a higher ^13^C abundance in the EPS fraction (∼60%) and a decrease of ^13^C abundance in the TLE (∼30%) in the mutant strain in comparison with the WT, although TLE always remained as the main cellular carbon fraction in those strains. Perry et al. (2007) reported the structural characterization of an EPS produced by *R. jostii* RHA1 as a high-molecular-mass polymer of a repeating tetrasaccharide unit composed of D-glucuronic acid, D-glucose, D-galactose, L-fucose, and an O-acetyl group (1:1:1:1:1). In this study, the EPS produced by the *nlpR*-disrupted mutant was also a sugar-rich polymer. Our previous results suggested that NlpR can be considered as a potent lipogenic factor, which regulates several genes involved in lipid and nitrogen metabolism (Hernández et al., 2017). In addition, this regulator might influence directly or indirectly the expression of other genes related to carbohydrate metabolism, including the EPS and glycogen biosynthesis. The role of NlpR in the carbon distribution to sugar-derivative compounds might involve additional regulatory proteins, which contain the non-canonical motif upstream of the respective genes (Hernández et al., 2017). On the other hand, NlpR might expand its influence on carbon distribution-related genes by a combined activity with the well-known global regulator GlnR. Amin et al. (2012) suggested that NlpR acts as a co-activator of GlnR for the expression of some genes of nitrogen metabolism in *Streptomyces coelicolor*. There is increasing evidence that the GlnR regulon is expanded to control carbon metabolism besides nitrogen metabolism, and particularly the carbohydrate metabolism in some actinobacteria (Liao et al., 2015). Thus, the *nlpR* mutation might produces an imbalance of GlnR/NlpR-mediated processes under nitrogen limitation, altering the carbon distribution to carbohydrate metabolism. Finally, changes in the EPS and glycogen biosynthesis might occur as a secondary effect of *nlpR* disruption due to the surplus of metabolites. In this context, *nlpR* disruption may induce an imbalance between glycolytic and central metabolic pathways, which can serve as a driving force for the biosynthesis of sugar-enriched products in the mutant. Further studies are necessary to determine the functional relation between NlpR and the carbohydrate metabolism.

The major impact of *nlpR* disruption was the decrease in the production rates of TLE and its fractions (NL, GL, and PL). Among these lipid fractions, NL was always the main labeled fraction at the expense of the TAG fraction (**Figure 4**). Lipid analyses demonstrated that besides TAG fraction, the absolute amounts of the DAG and FFA fractions were also decreased in mutant strain (**Figure 4**), but without significant changes in the FA composition profiles (**Figure 5** and Supplementary Figure S4). Thus, NlpR seems to modulate the FAS system only at a quantitative level. The most notorious effect of *nlpR* mutation was the significant decrease of ^13^C incorporation into the DAG fraction corrected by ^13^C TLE fraction (**Figure 4C**). Clearly, the labeled carbon distribution into DAG and TAG fractions within the TLE fraction was different in the *nlpR*-disrupted mutant as is shown in **Figure 4C**. It is known that DAG overaccumulation leads to cell dysfunction and for this reason, intracellular DAG levels are usually strictly regulated by different enzymes such as DAG-kinase, DAG-lipase, DGAT, PAP-2, among others. Thus, the lower ^13^C incorporation into the DAG fraction observed in mutant strain could be the result of a higher recycling process of this molecule, yielding in a label dilution of the sample. In any case, proper regulation of DAG is pivotal for cell homeostasis and our results suggested that the availability of DAG in cells is one of the critical control points for the regulation of lipogenesis in oleaginous rhodococci.

### Overexpression of *nlpR* under Nitrogen-Rich Conditions

We also analyzed the effect of *nlpR* overexpression on the distribution of the labeled carbon through the metabolism during cultivation of cells under nitrogen-rich conditions, in which NlpR is not naturally induced (Hernández et al., 2017). These culture conditions usually promote cell growth and biomass production, but not storage lipid accumulation. Thus, PL biosynthesis might be more stimulated than TAG production. In a previous study, we demonstrated that the expression of *nlpR* gene using an inducible promoter produced an increase of lipid production in *R. jostii* RHA1 as result of the modular expression of lipogenesis machinery components during an unfavorable condition for TAG accumulation (Hernández et al., 2017). In this study, *nlpR* overexpression in RHA1 cells under nitrogen-rich conditions resulted in an increase of the label incorporation into the TLE (**Figure 2**). The highest change of ^13^C in overexpressing cells was observed for the PL fraction (**Figures 3B,C**). Moreover, higher ^13^C enrichment occurred in TAG and FFA fractions after *nlpR* induction as revealed EA-IRMS analysis. Despite DAG fraction of overexpressing strain was missed in those experiments, GC-IRMS used as alternative approach revealed also a higher ^13^C enrichment of DAG fraction in overexpressing cells. Thus, overexpressing cells showed the opposite phenotype than that observed in mutant strain in regard to TAG, DAG, and FFA fractions, confirming the contribution of *nlpR* gene in the homeostasis of these compounds. In this context, we previously demonstrated that the *nlpR* disruption, affects the expression of genes associated with FASI and FASII, as well as other genes belonging to the Kennedy pathway necessary for the biosynthesis of TAG, DAG, and PL (Hernández et al., 2017). Results of this study indicate that the overexpression of *nlpR* gene during cells growth conditions (high nitrogen-media) reinforces the carbon flux to FAS, which are distributed not only into PL biosynthesis but also into TAG biosynthesis, which in turn are usually poorly synthesized under those conditions. It is important to consider that PL are also needed for the assembly of lipid inclusion bodies during TAG accumulation (Fujimoto et al., 2008; Chen et al., 2013).

## Conclusion

**FIGURE 6:**
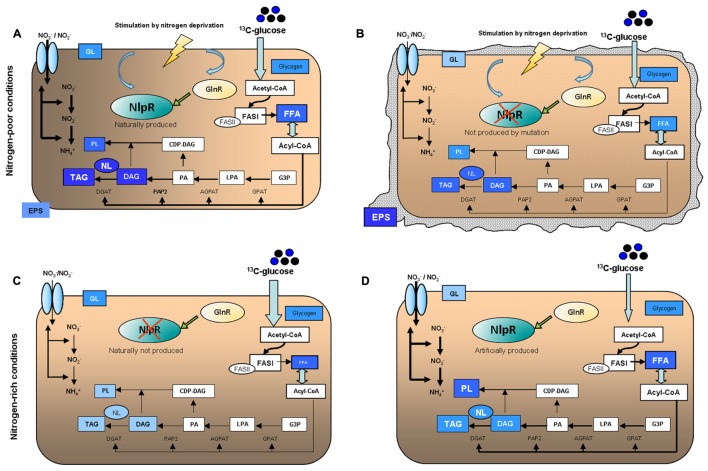
Schematic representation of main phenotypes occurring in **(A)** RHA1 WT, **(B)** RHA1::*nlpR*, cultured under nitrogen-poor conditions (MSM0.1), and **(C)** RHA1 pTipQC2, **(D)** RHA1 pTipQC2/*nlpR*, cultured under nitrogen-rich conditions (MSM1). The intensity of the metabolic flow is represented by the thickness of the lines or arrows. Studied compounds are shown in blue boxes, and the occurrence of a higher or lower labeling of the same is represented by combining the blue color intensity and the font size.

Our results indicate that certainly NlpR is not essential for lipogenesis and TAG accumulation, since *nlpR*-disrupted mutant still accumulated significant amounts of TAG. This suggests that additional regulatory components are simultaneously promoting lipogenesis and TAG accumulation in RHA1 cells during nitrogen limitation. However, NlpR provides a stronger redirection of carbon flux toward lipid metabolism, maximizing lipogenesis, and TAG accumulation specifically under N-limiting conditions. Thus, nitrogen limitation is the main trigger to NlpR activation, probably in orchestration and in response to other regulators such as the global regulator GlnR. A model summarizing the main phenotypes observed in this study is shown in **Figure 6**. An emerging model of the role of NlpR in overall metabolism indicates that this transcriptional factor behaves as a global regulator of nitrogen-mediated lipogenesis, contributing to the lipid homeostasis in oleaginous rhodococci and the allocation of carbon into different lipid fractions. Thus, TAG, DAG, FFA, and PL were the main lipid fractions altered in cells during modification of *nlpR* expression under nitrogen-limiting and nitrogen-rich conditions. The *nlpR* characterization provides evidence of an endogenous regulatory system which controls large metabolic modules involving lipogenesis in response to nutritional changes (nitrogen source) in the environment. The significant role of NlpR in the lipogenesis and TAG accumulation in *R. jostii* RHA1 indicates that this transcriptional regulator is a component of a complex regulatory network contributing to oleagenicity.

## Author Contributions

MH participated in the design of the study, carried out the experimental studies, and drafted the manuscript. DS participated in the design and analysis of lipid fractionation. GG participated in the coordination of the study and in the interpretation of data. HA conceived the study and participated in its design and coordination, interpretation of data, and helped to draft manuscript. All authors read and approved the final manuscript.

## Conflict of Interest Statement

The authors declare that the research was conducted in the absence of any commercial or financial relationships that could be construed as a potential conflict of interest.
