# Supplementary material for: Carbon Allocation in Rhodococcus jostii RHA1 in Response to Disruption and Overexpression of nlpR Regulatory Gene, Based on 13C-labeling Analysis
Source: Front Microbiol. 2017 Oct 11;8:1992. doi: 10.3389/fmicb.2017.01992 (PMC5641563; doi:10.3389/fmicb.2017.01992)
Supplement: Supplementary file 1 [file Data_Sheet_1.PDF]

Supplementary material

Carbon allocation in *Rhodococcus jostii* RHA1 in response to disruption and over-expression of *nlpR* regulatory gene, based on ^{13}C-labelling analysis

Martín A. Hernández[1], Gerd Gleixner[2*], Dirk Sachse[3, 4], Héctor M. Alvarez[1*]

[1]Instituto de Biociencias de la Patagonia - Consejo Nacional de Investigaciones Científicas y Técnicas, Facultad de Ciencias Naturales, Universidad Nacional de la Patagonia San Juan Bosco, Comodoro Rivadavia, Chubut, Argentina.
[2]Department of Biogeochemical Processes, Max Planck Institute for Biogeochemistry Jena, Germany.
[3]GFZ German Research Centre for Geosciences, Section 5.1 Geomorphology, Potsdam, Germany
[4]Institute of Earth and Environmental Science, University of Potsdam, Potsdam, Germany.

Correspondence:

Héctor M. Alvarez: halvarez@unpata.edu.ar

Gerd Gleixner: ggleix@bgc-jena.mpg.de

Supplementary figures legends

Figure S1: Qualitative TLC analysis of extracted lipid fractions from **1.** RHA1 WT, **2.** RHA1::*nlpR*, **3.** RHA1pTipQC2 and **4.** RHA1 pTipQC2/*nlpR*. **(A)** Analysis of neutral lipids (NL), glycolipids (GL) and phospholipids (PL) obtained by solid-phase extraction (SPE) column chromatography with unmodified silica stationary phase. **(B)** Analysis of F1 to F5 obtained by solid-phase extraction (SPE) column chromatography with amino propyl stationary phase. Solvents used for elution of several fractions are indicated. **(C)** Fractionation steps of F3 of each strain for TAG and DAG separation.

Figure S2: Cellular growth profiles and total glucose (unlabelled+labelled) consumption of analyzed strains of this study. The point where cells were harvested for ^{13}C analysis is indicated by an arrow.

Figure S3: Total ^{13}C fatty acid content measured by GC-IRMS in **(A**) Neutral lipids, **(B)** Phospholipids, **(C)** triacylglycerols, **(D)** diacylglycerols and **E)** free fatty acids of RHA1 derivative strains. Values are expressed as % of ^{13}C content by cellular dry weight (CDW).

Figure S4: Fatty profiles and ^{13}C fatty acid absolute concentration measured by GC-IRMS in **(A**) Neutral lipids, **(B)** Phospholipids, **(C)** triacylglycerols, **(D)** diacylglycerols and **(E)** free fatty acids of RHA1 derivative strains.

Fig. S1:

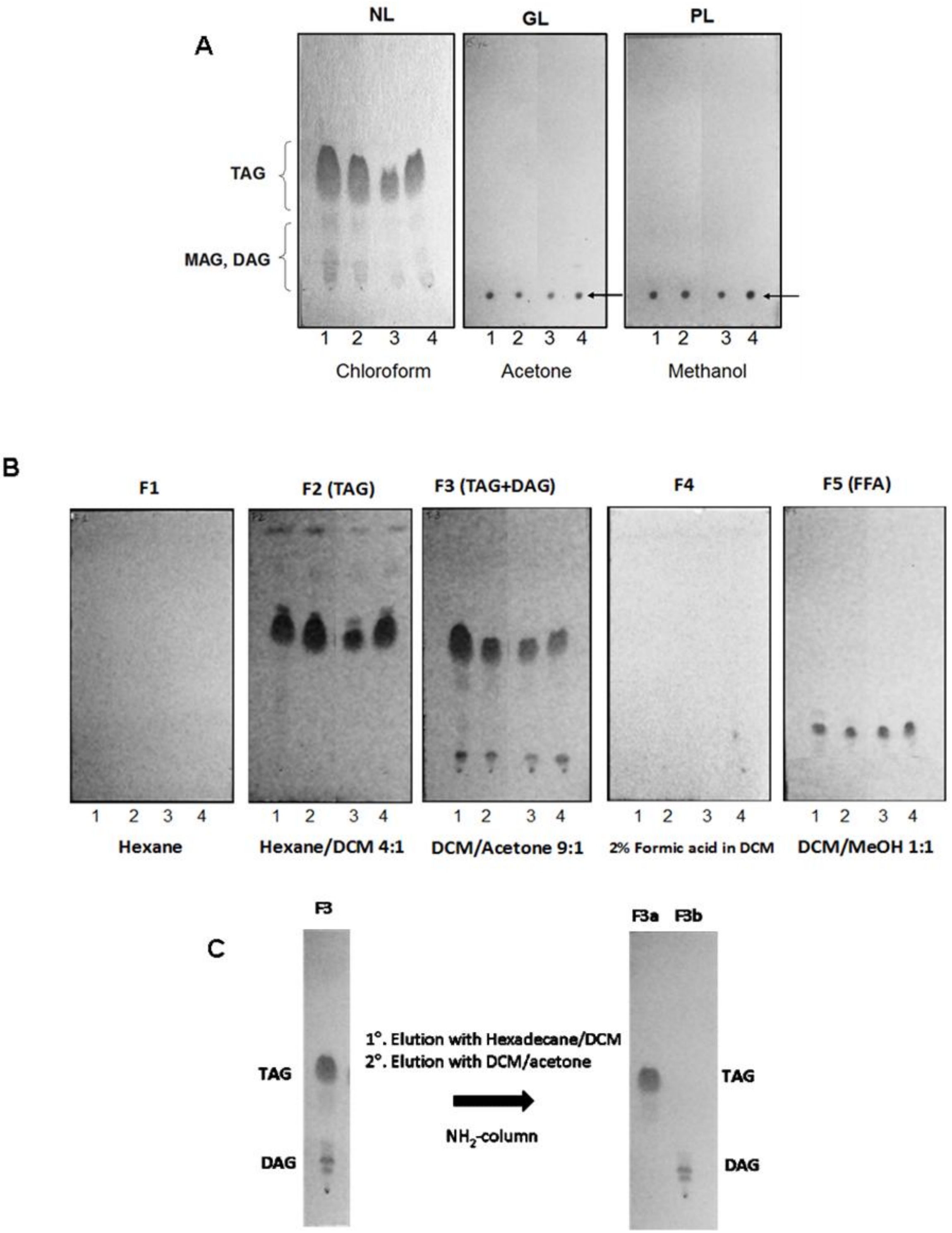
A
NL
GL
PL
TAG
MAG, DAG
1 2 3 4
Chloroform
Acetone
Methanol
B
F1
F2 (TAG)
F3 (TAG+DAG)
F4
F5 (FFA)
Hexane
Hexane/DCM 4:1
DCM/Acetone 9:1
2% Formic acid in DCM
DCM/MeOH 1:1
C
F3
F3a F3b
TAG
DAG
1°. Elution with Hexadecane/DCM
2°. Elution with DCM/acetone
NH_2-column

Fig. S2:

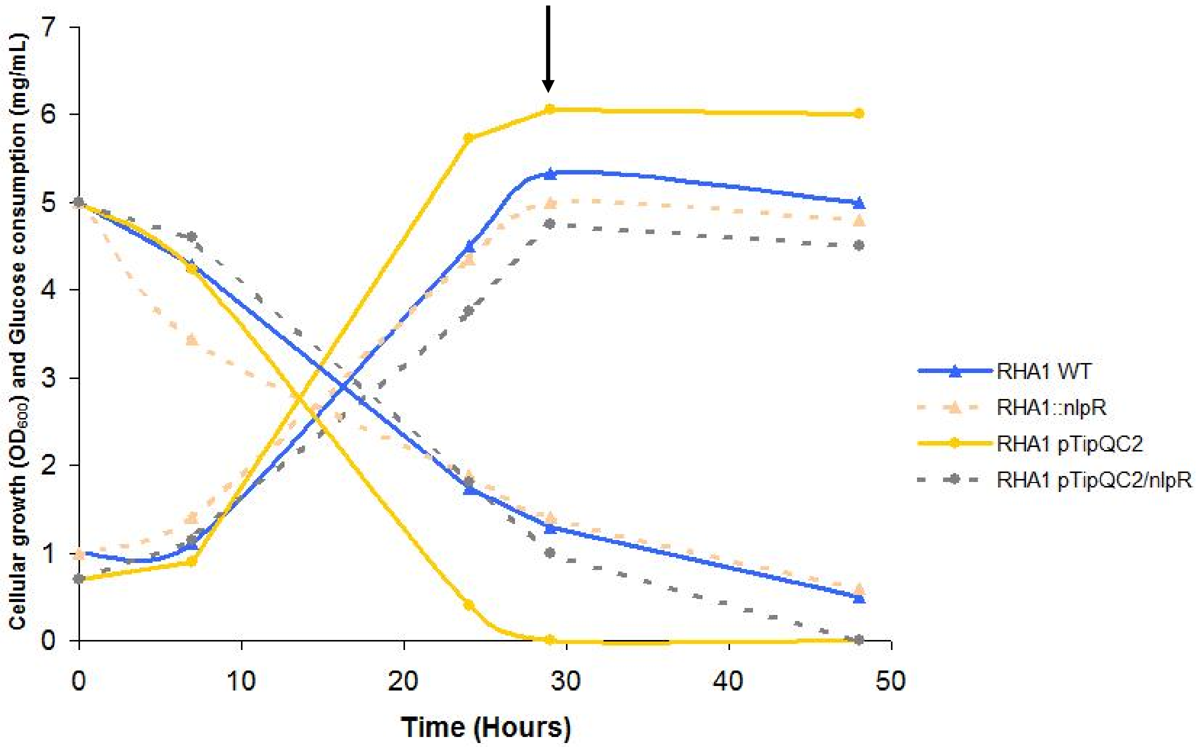

Cellular growth (OD600) and Glucose consumption (mg/mL)
Time (Hours)
RHA1 WT
RHA1::nlpR
RHA1 pTipQC2
RHA1 pTipQC2/nlpR
0
1
2
3
4
5
6
7
0
10
20
30
40
50

Fig. S3:

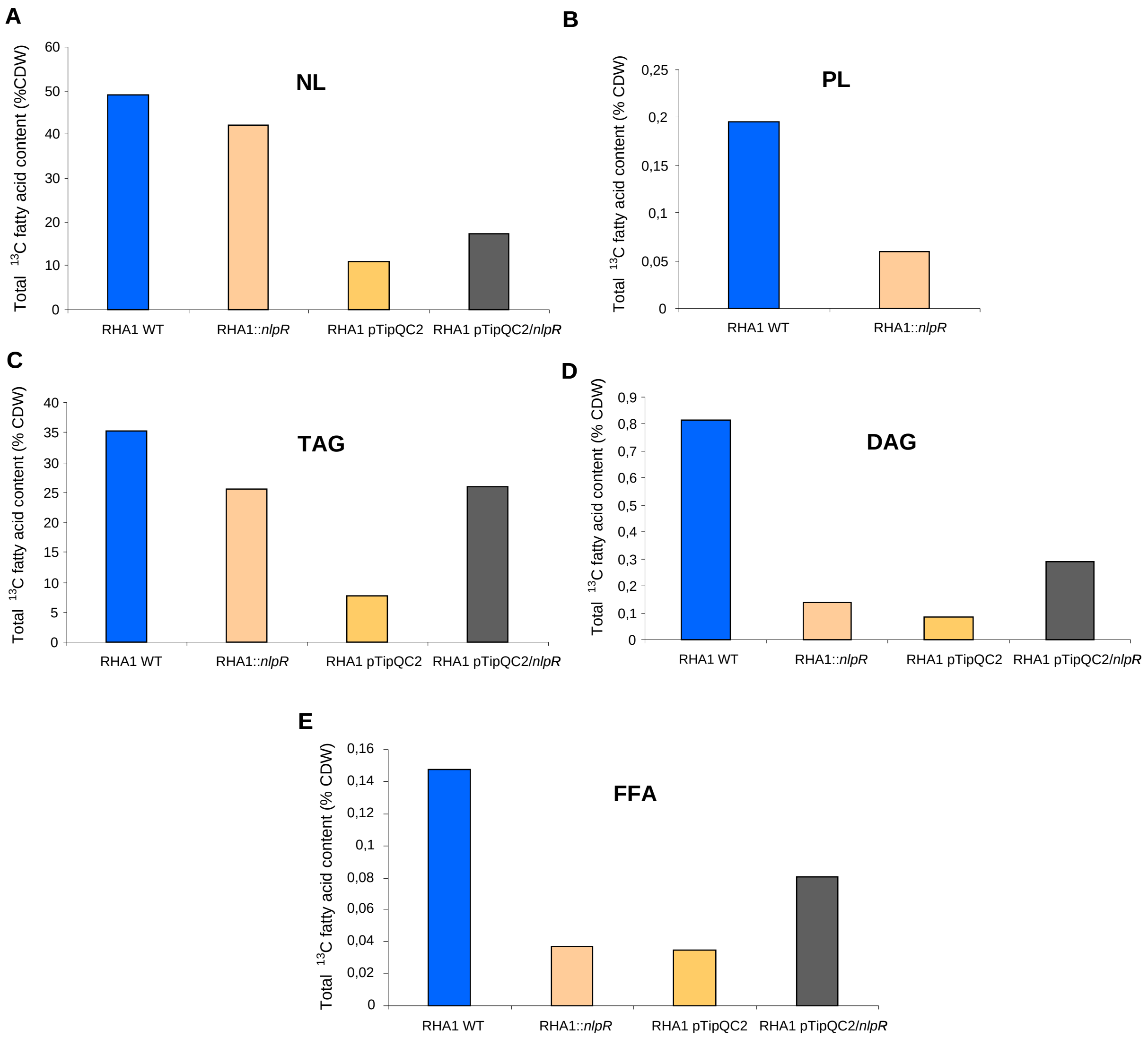
A
NL
Total ^{13}C fatty acid content (%CDW)
60
50
40
30
20
10
0
RHA1 WT
RHA1::*nlpR*
RHA1 pTipQC2
RHA1 pTipQC2/*nlpR*
B
PL
Total ^{13}C fatty acid content (% CDW)
0,25
0,2
0,15
0,1
0,05
0
RHA1 WT
RHA1::*nlpR*
C
TAG
Total ^{13}C fatty acid content (% CDW)
40
35
30
25
20
15
10
5
0
RHA1 WT
RHA1::*nlpR*
RHA1 pTipQC2
RHA1 pTipQC2/*nlpR*
D
DAG
Total ^{13}C fatty acid content (% CDW)
0,9
0,8
0,7
0,6
0,5
0,4
0,3
0,2
0,1
0
RHA1 WT
RHA1::*nlpR*
RHA1 pTipQC2
RHA1 pTipQC2/*nlpR*
E
FFA
Total ^{13}C fatty acid content (% CDW)
0,16
0,14
0,12
0,1
0,08
0,06
0,04
0,02
0
RHA1 WT
RHA1::*nlpR*
RHA1 pTipQC2
RHA1 pTipQC2/*nlpR*

Fig. S4:

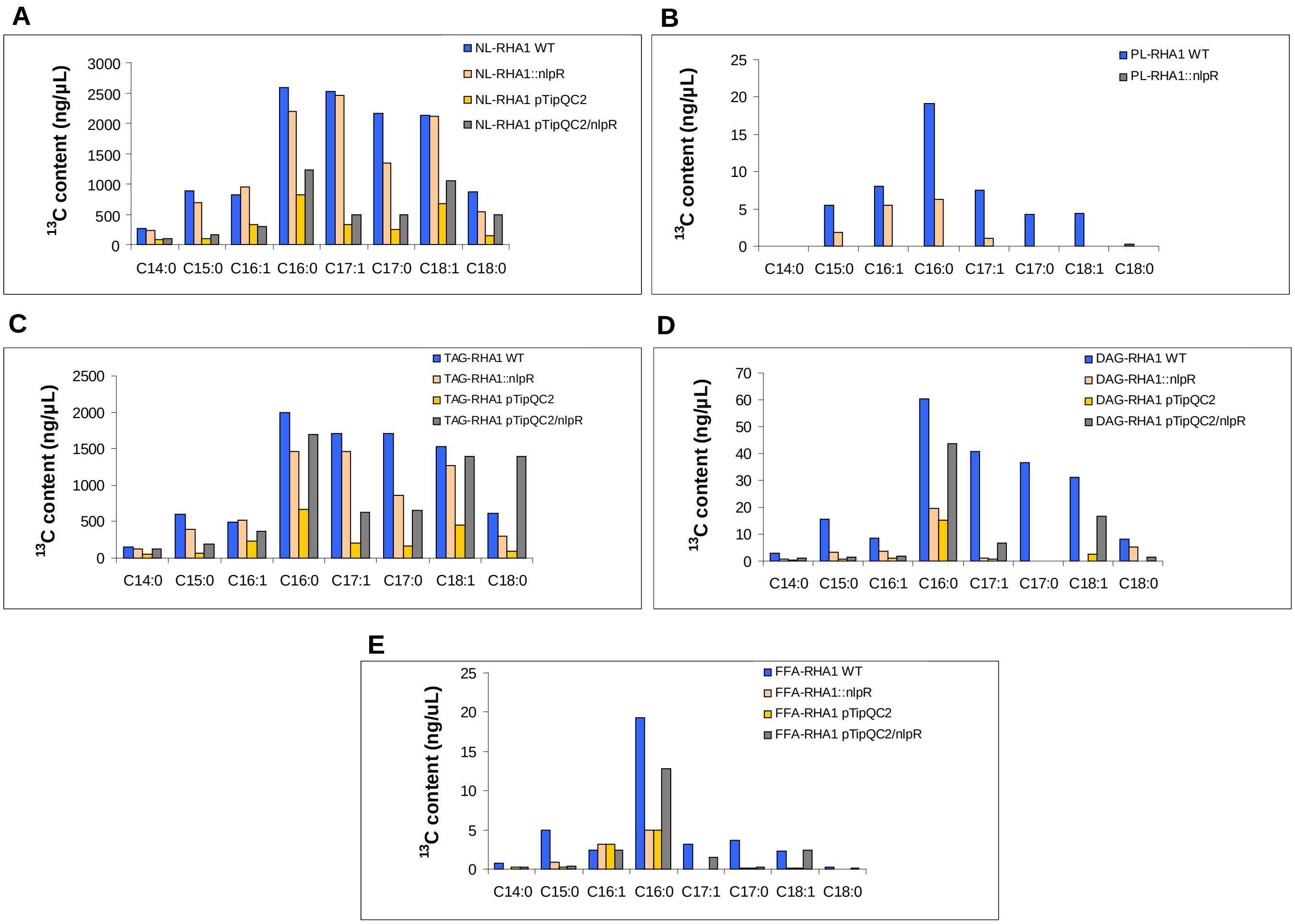
A
NL-RHA1 WT
NL-RHA1::nlpR
NL-RHA1 pTipQC2
NL-RHA1 pTipQC2/nlpR
^{13}C content (ng/μL)
0 500 1000 1500 2000 2500 3000
C14:0 C15:0 C16:1 C16:0 C17:1 C17:0 C18:1 C18:0
B
PL-RHA1 WT
PL-RHA1::nlpR
^{13}C content (ng/μL)
0 5 10 15 20 25
C14:0 C15:0 C16:1 C16:0 C17:1 C17:0 C18:1 C18:0
C
TAG-RHA1 WT
TAG-RHA1::nlpR
TAG-RHA1 pTipQC2
TAG-RHA1 pTipQC2/nlpR
^{13}C content (ng/μL)
0 500 1000 1500 2000 2500
C14:0 C15:0 C16:1 C16:0 C17:1 C17:0 C18:1 C18:0
D
DAG-RHA1 WT
DAG-RHA1::nlpR
DAG-RHA1 pTipQC2
DAG-RHA1 pTipQC2/nlpR
^{13}C content (ng/μL)
0 10 20 30 40 50 60 70
C14:0 C15:0 C16:1 C16:0 C17:1 C17:0 C18:1 C18:0
E
FFA-RHA1 WT
FFA-RHA1::nlpR
FFA-RHA1 pTipQC2
FFA-RHA1 pTipQC2/nlpR
^{13}C content (ng/uL)
0 5 10 15 20 25
C14:0 C15:0 C16:1 C16:0 C17:1 C17:0 C18:1 C18:0
